# Frequent central nervous system, pachymeningeal and plexus MRI changes in POEMS syndrome

**DOI:** 10.1007/s00415-019-09233-z

**Published:** 2019-02-12

**Authors:** Oliver J. Ziff, Chandrashekar Hoskote, Stephen Keddie, Shirley D’Sa, Indran Davangnanam, Michael P. T. Lunn

**Affiliations:** 1 MRC Centre for Neuromuscular Diseases, National Hospital for Neurology and Neurosurgery, London, UK; 2 Department of Molecular Neuroscience, UCL Institute of Neurology, London, UK; 3 Lysholm Department of Neuroradiology, National Hospital for Neurology and Neurosurgery, London, UK; 4 Cancer Division, Department of Haematology, University College London Hospitals NHS Foundation Trust, London, UK; 5 Department of Neuroimmunology, The National Hospital for Neurology and Neurosurgery, UCL Institute of Neurology, Queen Square, London, WC1N 3BG UK

**Keywords:** POEMS, Magnetic resonance imaging, Pachymeningitis, Pachymeningeal thickening

## Abstract

**Objective:**

Polyneuropathy, organomegaly, endocrinopathy, monoclonal gammopathy, skin changes (POEMS) syndrome is a rare multisystem disease associated with a plasma-cell dyscrasia. Although pachymeningeal involvement has occasionally been described, MRI of the central nervous system (CNS) has not yet been extensively investigated.

**Methods:**

We retrospectively evaluated CNS MRI in Europe’s largest single-center cohort of POEMS syndrome. Of 77 patients who have been formally diagnosed with POEMS, 41 had MRI brain and 29 had MRI spine. A control group of 33 patients with chronic inflammatory demyelinating polyneuropathy (CIDP) was used as this is the major differential diagnosis. Of these CIDP patients, 12 underwent both MRI brain and spine, 7 had solely MRI brain and 14 had MRI spine.

**Results:**

In 41 POEMS patients with MRI brain, we identified frequent smooth, diffuse meningeal thickening of the cerebral convexities and falx (*n* = 29, 71%), of which 4 had meningeal collections. 17 (41%) had vascular abnormalities including white-matter disease, of which 4 had established infarcts. Of 29 patients with MRI spine, 17 (59%) had thickening of the brachial and lumbosacral plexus. Conversely in 19 CIDP patients with MRI brain, none had meningeal thickening (*p* < 0.0001); however, 8 (42%) had vascular abnormalities (*p* = 0.85). Of 26 patients with MRI spine, 9 (35%) had brachial or lumbosacral plexus thickening (*p* = 0.06).

**Conclusions:**

In contrast to CIDP, POEMS patients frequently have pachymeningeal thickening. Vascular abnormalities and plexus thickening were also common but not statistically different to CIDP.

**Electronic supplementary material:**

The online version of this article (10.1007/s00415-019-09233-z) contains supplementary material, which is available to authorized users.

## Introduction

POEMS (polyneuropathy, organomegaly, endocrinopathy, monoclonal plasma cell disorder, skin changes) syndrome is a rare and disabling multisystem condition characterised by peripheral neuropathy and a monoclonal plasma cell disorder [1]. Additional manifestations include papilloedema, extravascular volume overload, sclerotic bone lesions and thrombocytosis. However, clinical presentations are heterogeneous making the diagnosis challenging. To standardise the diagnosis, the major and minor diagnostic criteria were proposed in 2003 [5] but despite this POEMS remains diagnostically elusive. Patients commonly present with only a limited number of the criteria and the subtle bone marrow abnormalities provide false reassurance regarding the plasma cell dyscrasia.

Patients are frequently (~ 60%) misdiagnosed with other neuropathies, usually chronic inflammatory demyelinating polyradiculoneuropathy (CIDP), and subsequently receive ineffective immunomodulatory medications [8, 9]. CIDP is more common and the neuropathy alone can be difficult to distinguish. Furthermore, electrical studies reveal conduction slowing in both, often with no specific features to discriminate one from the other. Relevant biochemical markers of POEMS include lambda light chain associated monoclonal gammopathies and VEGF [8]. Although current recommendations advise imaging for bone lesions, no guidance exists on imaging the central nervous system (CNS) [10]. Identifying disease-specific characteristics through detailed phenotyping will assist in improving both the sensitivity and specificity of current diagnostic criteria. Although CNS abnormalities including pachymeningeal involvement have been described in small case series [2, 3, 11], CNS MRI has not been extensively investigated. This study systematically reviews all our CNS-imaged patients with a confirmed POEMS diagnosis and compares these findings with a CIDP control group. Specifically, we investigated the frequency of meningeal thickening and enhancement, explored CNS parenchymal abnormalities and examined nerve roots and plexi.

## Methods

### Study design and participants

This study was performed at the POEMS Clinic at University College London Hospital, which treats the largest reported POEMS cohorts in Europe. 77 patients who have attended this clinic from January 2010 to August 2018 have been formally diagnosed with POEMS syndrome according to the current diagnostic criteria [5]. Serum VEGF, immunoglobulins, serum-free light chains, bone marrow biopsy and nerve condition studies were collected from all patients. 43 patients underwent lumbar puncture and cerebrospinal fluid (CSF) was tested for tuberculosis and viral studies including herpes and enterovirus. Symptom duration was calculated as the time interval between symptom onset and MRI date. 41 patients with POEMS (25 male [61.0%], mean age 60 years [range 33–79 years]) underwent brain MRI and 29 spine MRI. MRI was performed before the diagnosis of POEMS was confirmed in 24 of 41 (58.5%) patients (Supplementary Table 1). 33 age- and sex-matched patients from the University College London Hospital CIDP database were used as controls, with 19 having brain MRI and 26 having spine MRI available (Supplementary Table 2).

### Neuroradiological assessment

Patients underwent MRI with gadolinium using a 3.0T MRI scanner with a 32-channel receive coil. In our institution, the routine MRI protocol to assess for meningeal disease includes both axial and coronal pre- and post-contrast FLAIR, sagittal T1-weighted images (including diffusion weighted) and axial T2-weighted images. FLAIR imaging was performed using a fast FLAIR sequence with a repetition time/effective echo time/inversion time of 9000/150/2400 ms. T1-weighted imaging was performed using the 2-dimensional spin-echo method (TR, TE: 500, 8). All imaging sequences were acquired with a slice thickness/interslice gap = 5/1 mm. Meningeal thickness was measured at the point of maximal thickness with > 1 mm taken as abnormal. To evaluate nerve roots and plexi, spine images including coronal gadolinium-enhanced fat-suppressed T1-weighted, coronal short-tau inversion recovery, and axial fat-saturated T2-weighted spin-echo sequences were obtained. Post-contrast images were obtained after intravenous injection of 0.1 mmol/kg gadopentetate dimeglumine (gadolinium—GAD). Post-contrast FLAIR imaging was performed prior to post-contrast T1-weighted imaging in all cases. Images were independently assessed by two consultant neuroradiologists (I.D., C.H.), each with > 10 years of specialist experience, blinded to the diagnosis and scans were acquired over an 11 year period (2007–2018). Images were viewed using PACS (Agfa) IMPAX 6.5.1 on 3-megapixel BARCO monitors.

No patients had received treatment for POEMS at the time of MRI; however, 66% were previously misdiagnosed with CIDP and had received intravenous immunoglobulin therapy. As neuroradiological findings are not currently part of the POEMS diagnostic criteria, MRI was not used to contribute to the POEMS diagnosis in any patients. Clinical MRI indications varied but MRI was not part of an algorithm for the diagnosis of POEMS. Spinal and plexus MRI were performed as part of the diagnostic workup for demyelinating neuropathies, predominantly CIDP. Brain MRI was performed directed at focal CNS symptoms or signs, including strokes, to assist in therapeutic anticoagulation decisions and also for non-specific symptoms (headache). Occasionally it had been performed as part of a ‘whole neuroaxis’ protocol where even in peripheral nerve disorders MRI often gets performed by referring teams for ‘limb weakness’ without any obvious CNS signs. Due to insufficient repeat MRI scans available, we were unable to follow CNS changes over time and in response to chemotherapy or stem cell transplantation. After MRI, most patients (57%) underwent a contemporaneous lumbar puncture for cerebrospinal fluid analysis, although they may have had an LP many months or years beforehand from which any residual meningeal change would have resolved.

### Statistical analysis

Mean, standard deviation and standard error were calculated for all variables. Pearson’s correlation coefficient and stepwise linear regression analysis were used to compare serum biomarkers and symptom duration with neuroradiological findings. Fisher’s exact test was used to compare findings between POEMS and CIDP groups. A p value of < 0.05 was considered statistically significant. Analyses were performed using STATA version 13.1 (StataCorp LP, Texas).

### Ethical and data availability statement

Ethical approval was not required for this fully anonymised and retrospective observational (non-experimental) study. All anonymised individual participant data in this retrospective cohort study are available in the Supplementary Table.

## Results

At the time of MRI, the mean age was 59.5 years; 61% were male and 67% were white British and the mean disease duration was 35 months. Mean pre-treatment VEGF at diagnosis was 3917 pg/mL (range 200–27,310 pg/mL). CSF protein was abnormally raised with mean 1.75 g/L (range 0.6–4.8 g/L). Neurophysiological data in POEMS were broadly homogeneous, with reports of sensory (84%) and motor (92%) neuropathy with demyelinating features (86%) and secondary axonal loss (75%). All studied patients had stable neurological findings at the time of MRI, except for one patient with headache and hypertension who had radiographic features of posterior reversible encephalopathy syndrome. Detailed neuroimaging data are summarised in the supplementary table.

### Brain MRI

**Fig. 1:**
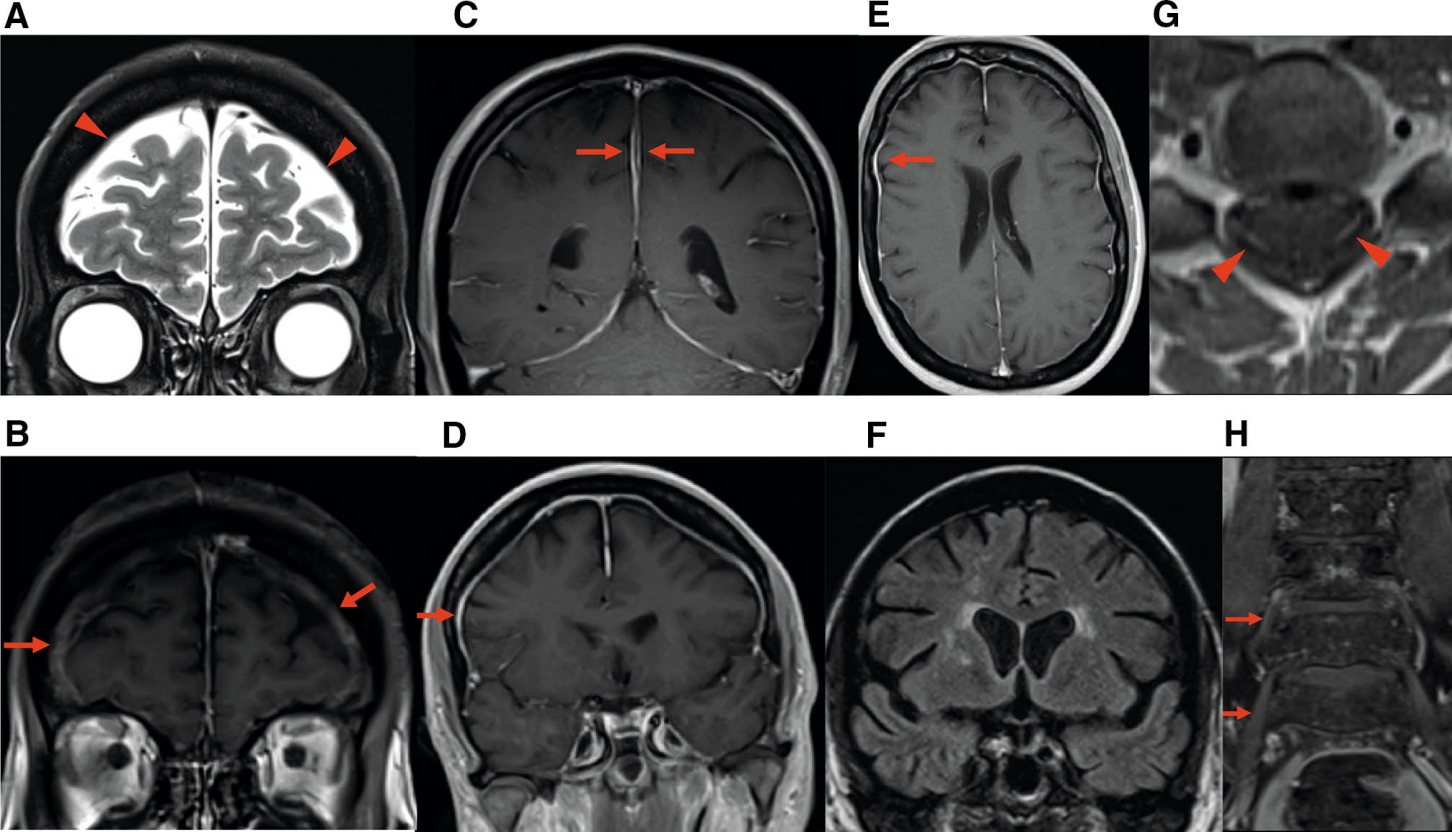
**a** Coronal T2w image showing apparent widening of subdural planes over bifrontal convexities. **b** Coronal post-gadolinium T1w equivalent plane image from the same patient demonstrating thick enhancing dura in this area. **c** Post-gadolinium T1w coronal image showing two opposing layers of enhancing dura. The central non-enhancing area is effusion: the “double falx” appearance. **d** Post-gadolinium T1w image demonstrating diffuse enhancement of intracranial pachymeninges. **e** Post-gadolinium T1w image demonstrating focal enhancement of intracranial pachymeninges. **f** Coronal FLAIR showing moderate degree of small vessel disease. **g** Post-gadolinium T1w image of the cervical spine level C5 showing intradural leptomeningeal enhancement of cervical nerve roots. **h** Thickened and enhancing roots/proximal lumbar plexus on post-gadolinium T1w coronal image. For further images and sequences, please see the Supplementary Image Series

In the POEMS cohort, 29 of 41 patients (70.7%) had hypertrophic pachymeningeal involvement. There was diffuse dural thickening involving the majority of the cranial convexities in 27 cases with localised thickening identified in 2 patients; thickening was smooth in all cases (see Fig. 1 and Supplementary Image Series). T1-weighted images revealed the thickened areas to be hypointense compared to the subjacent brain parenchyma and gadolinium sequences revealed dural enhancement. Maximal dural thickness varied with mean 2.1 mm (range 1–5 mm) with the area of maximal thickness most often being in the frontal region. In four cases, prominent meningeal collections were noted involving the falx cerebri. In all four cases, the imaging features of these collections were consistent with free fluid, with high signal on T2, low signal on T1, suppressing on FLAIR and non-enhancing on post-gadolinium studies. The abnormality was evident in all in the coronal plane images (see Supplementary Image Series). There were no other radiographic features of a low-pressure CSF state to explain the pachymeningeal involvement, including tonsillar displacement, brainstem slumping, venous distension, pituitary gland size (mean 3.5 ± 1.4 mm), subdural effusions or reduced CSF volume. None of the 19 CIDP patients with MRI brain had meningeal thickening (*p* < 0.0001 vs. POEMS).

White matter abnormalities consistent with small vessel disease were found in 17 of 41 POEMS patients (41%). Four of these patients (10%) had established infarcts. Comparatively, eight of 19 (42%) CIDP patients had white matter abnormalities also consistent with small vessel disease (*p* = 0.85 vs. POEMS).

Neither meningeal thickness or vascular abnormalities were correlated with VEGF (*r* = 0.05, *p* = 0.78; *r* = 0.02, *p* = 0.89, respectively) or age (*r* = 0.33, *p* = 0.06; *r* = 0.18, *p* = 0.33). Although meningeal thickness was not correlated to symptom duration (*r* = 0.18, *p* = 0.26), a significant association was identified between vascular abnormalities and symptom duration (*r* = 0.50, *p* = 0.002).

### Spine MRI

17 of 29 POEMS patients (58.6%) with root and plexus imaging had thickening and enhancement of nerve roots. This involved both the brachial and lumbosacral plexi in 15 cases and the brachial plexus only in the remaining two patients. In four cases, the thickening involved the intradural lumbar nerve roots. No spinal cord parenchymal abnormalities were identified. Nine of 26 (34.6%) CIDP patients had thickening and enhancement of nerve roots (*p* = 0.06 vs. POEMS), involving the brachial plexus in 7, lumbosacral plexus in 1 and both the brachial and lumbosacral plexi in the remaining 1 patient.

## Discussion

This is the largest MRI assessment of the CNS, meninges and plexi in POEMS syndrome to date. Our major finding is that asymptomatic pachymeningeal involvement is a very common feature in POEMS syndrome being present in 70.7% of our imaged patients. The pachymeningeal thickening was asymptomatic and unrelated to disease duration, but also had an unusual distribution, especially in the falx cerebri, and was probably related to intrameningeal fluid effusion as illustrated in 4 cases by high field MRI. Other small case series (~ 10 patients) have identified pachymeningeal involvement at similar frequencies to this larger unselected cohort [2, 3, 11]. Interestingly, we also found frequent white matter abnormalities and evidence of established cerebral infarction at much higher rates than previously reported, although the prevalence was similar in the CIDP cohort [6]. We also demonstrate prevalent thickened and enhancing nerve roots of the brachial and lumbosacral plexi making distinction from CIDP by MRI difficult.

Cranial pachymeningeal involvement is rare. It can be idiopathic or secondary to a broad variety of conditions and imaging features have not been found to be specific to any aetiology [7]. Established aetiologies include chronic inflammatory and infectious disease (for example, sarcoid and tuberculosis), meningeal lymphomatosis and intracranial hypotension (but where leptomeningeal enhancement is more common). In the 57% of cases where lumbar puncture was performed, it was undertaken after MRI and inflammatory and infectious causes were excluded on CSF as well as blood testing. Pachymeningeal histopathological assessment in POEMS has previously demonstrated the absence of inflammatory changes, without evidence of fibrosis or cellular infiltration on histopathology, triggering the term POEMS-associated pachymeningeal involvement rather than pachymeningitis [2, 3]. Strong co-expression of VEGF and VEGF2 on the cellular membrane of meningothelial cells suggested that VEGF may be directly implicated in pachymeningeal pathogenesis [2].

The majority of POEMS patients (~ 60%) are misdiagnosed as having CIDP, leading to diagnosis delay, inefficient treatment and worsening mobility. It appears that pachymeningeal involvement is a particularly useful finding which distinguishes POEMS syndrome from CIDP. Current diagnostic criteria for POEMS syndrome are extensive and clinically comprehensive but do not include neuroimaging features. We found no imaging features of the vascular abnormalities or plexus thickening that differentiate POEMS from CIDP and, therefore, make these less useful in this instance. To improve diagnostic speed and accuracy, the POEMS diagnostic criteria may benefit from the addition of pachymeningeal thickening as a minor criterion; however, this requires further validation in a prospective and possibly independent analysis with a prospectively collected group of haematological disease controls (Table 1).

**Table 1:** Diagnostic criteria for POEMS Syndrome

Major criteria (Mandatory)	1. Polyneuropathy
	2. Monoclonal plasma cell proliferative disorder
Other Major criteria (one required)	3. Castleman disease
	4. Sclerotic bone lesions
	5. Raised vascular endothelial growth factor
Minor criteria	6. Organomegaly (spleen/liver/lymph nodes)
	7. Extravascular volume overload
	8. Endocrinopathy (adrenal, thyroid, pituitary, gonadal, parathyroid, pancreatic)
	9. Skin changes
	10. Papilloedema
	11. Thrombocytosis/polycythemia
Other useful features	Clubbing, weight loss, hyperhidrosis, pulmonary hypertension/ restrictive lung disease, thrombotic diathesis, diarrhoea, low vitamin B_12_

From Dispenzieri et al. [4] Results from this retrospective cohort study suggest that addition of “Pachymeningeal Thickening” as a minor criterion may aid diagnosis of POEMS syndrome, particularly in differentiation from CIDP. This requires future validation in a prospective longitudinal analysis

## Conclusions

Pachymeningeal thickening is highly prevalent in POEMS syndrome and helps distinguish from the major differential diagnosis, CIDP. Although both vascular abnormalities and thickened enhancing plexi were also observed in POEMS syndrome, these were at rates similar to CIDP.

## Electronic supplementary material

Below is the link to the electronic supplementary material.

Supplementary material 1 (PDF 22505 KB)

Supplementary material 2 (DOCX 31 KB)
