# Supplementary material for: Frequent central nervous system, pachymeningeal and plexus MRI changes in POEMS syndrome
Source: J Neurol. 2019 Feb 12;266(5):1067–72. doi: 10.1007/s00415-019-09233-z (PMC6469836; doi:10.1007/s00415-019-09233-z)
Supplement: Supplementary file 1 — Supplementary material 1 (PDF 22505 KB) [file 415_2019_9233_MOESM1_ESM.pdf]

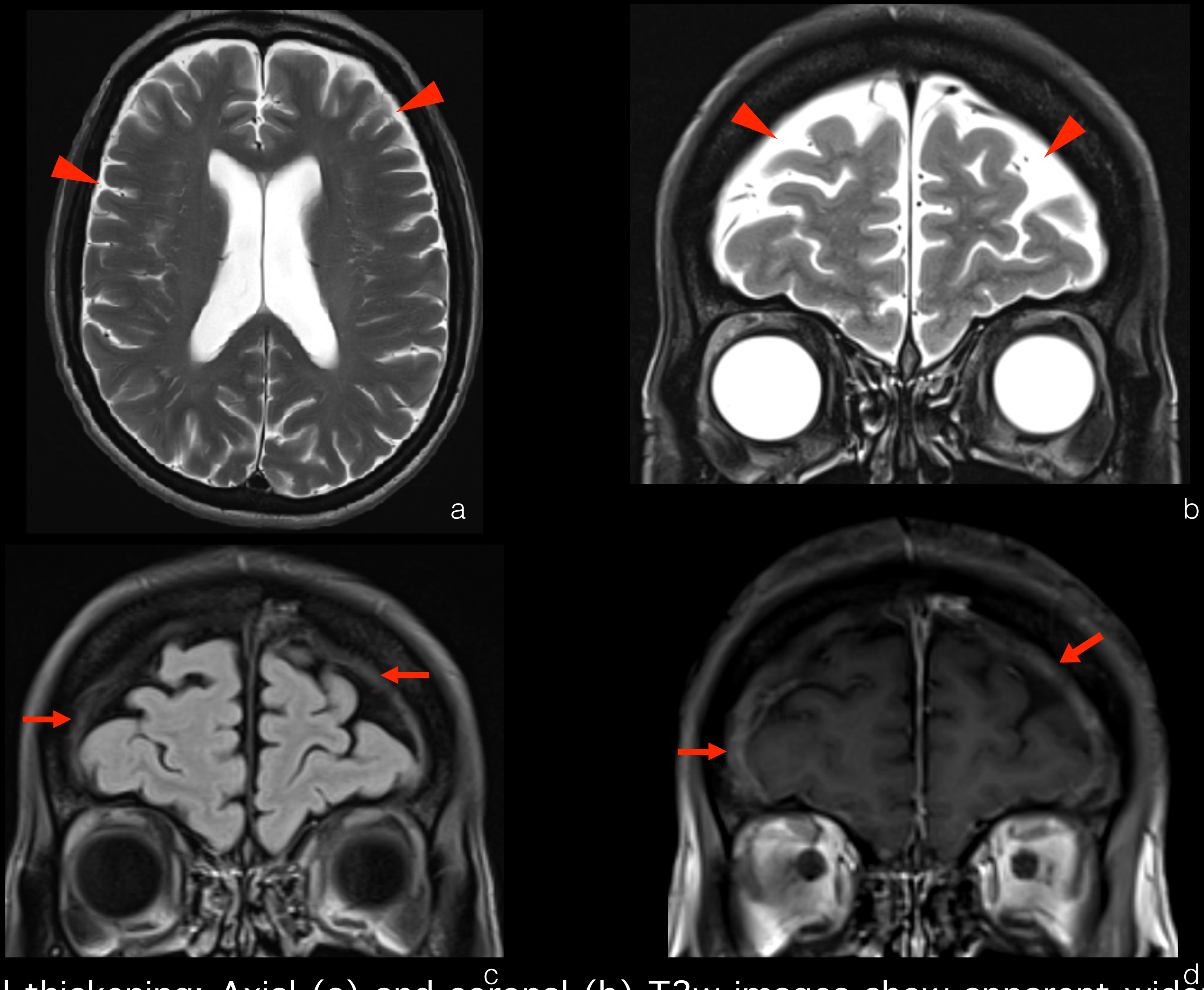

Pachymeningeal thickening: Axial (a) and coronal (b) T2w images show apparent widening of subdural planes over bifrontal convexities (arrow heads). Coronal FLAIR (c) and contrast-enhanced post-gadolinium T1w (d) images demonstrate thick, hyperintense, enhancing dura (arrows). All 4 images are from the same patient and are an extension of the main figure panel images A & B.

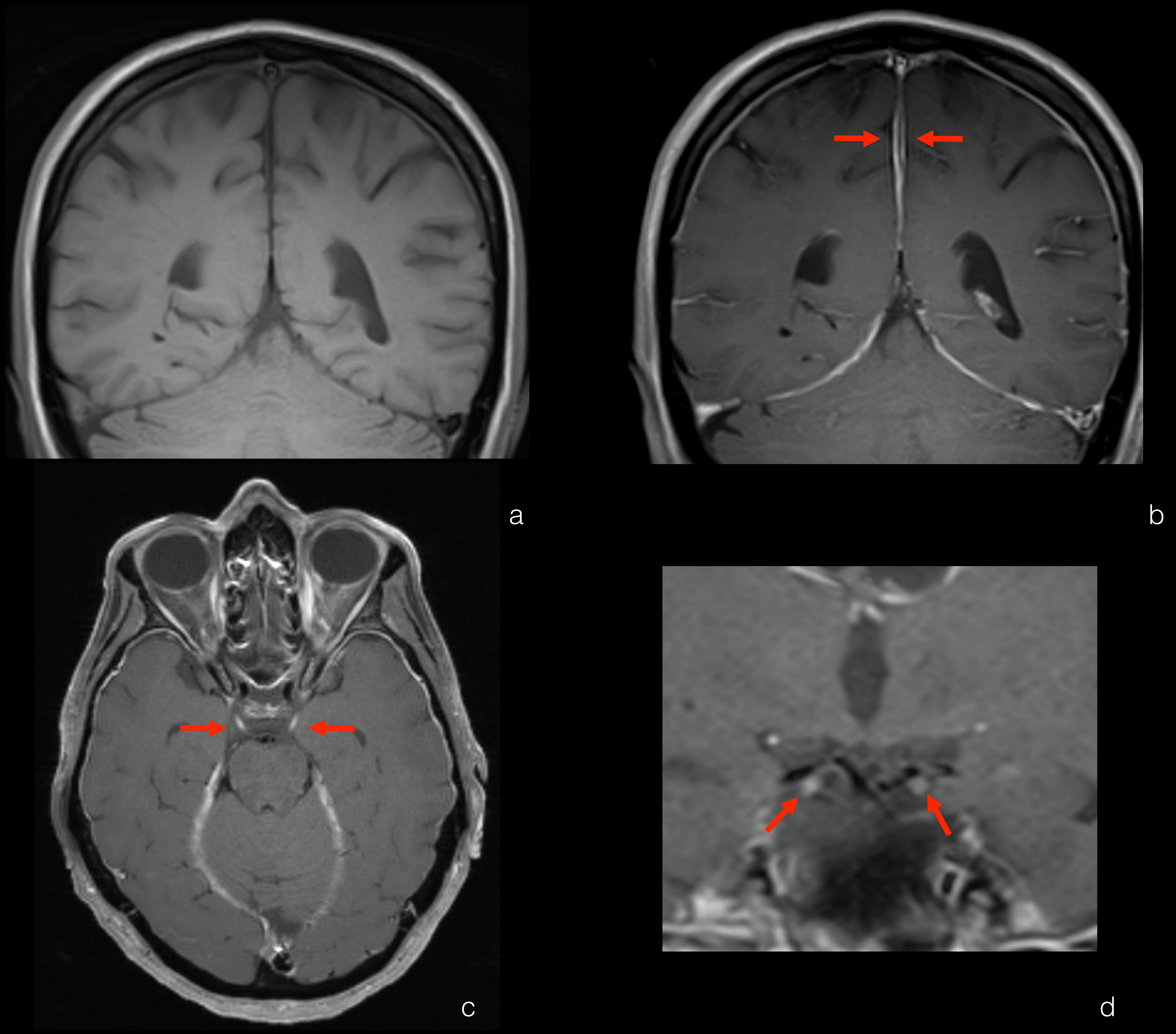

Pre (a) and post (b) gadolinium T1w coronal images show two opposing layers of enhancing dura. The central non-enhancing area is effusion: the ‘double falx’ appearance. Enhancement of the oculomotor nerves (arrows) in axial (c) and coronal (d) images of the same patient

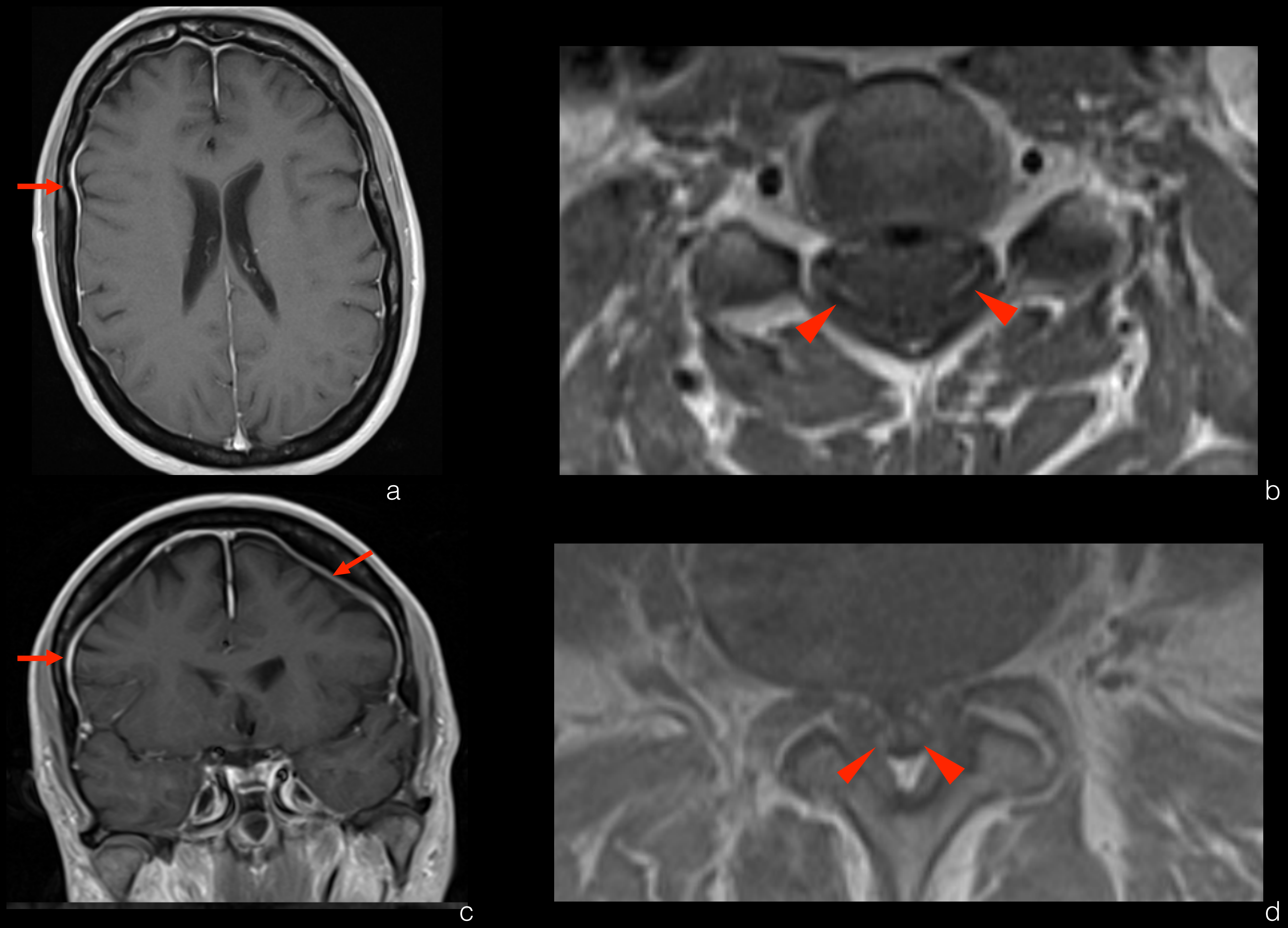

Post gadolinium T1w images of the brain (a,c), cervical (b) and lumbar (d) spine demonstrate enhancement of the intracranial pachymeninges (arrows) and intradural leptomeninges (arrowheads) of cervical and lumbar roots.

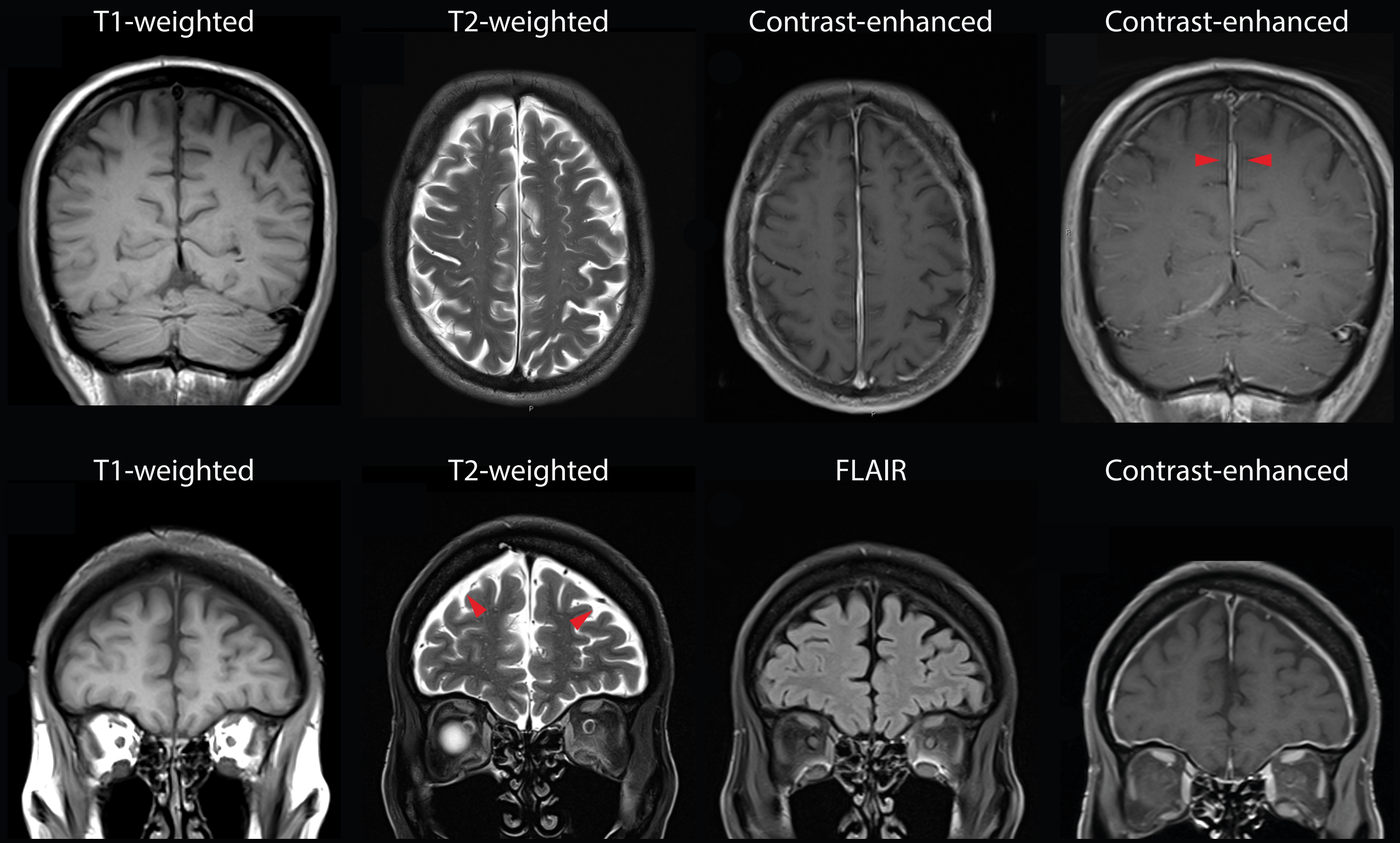

Axial and coronal planes of T1-weighted, T2-weighted, FLAIR and contrast-enhanced images from the same patient with intermeningeal fluid effusion (arrowheads). Fluid is identified as low signal on T1, high signal on T2, supressing on FLAIR and non-enhancing on post-gadolinium studies.

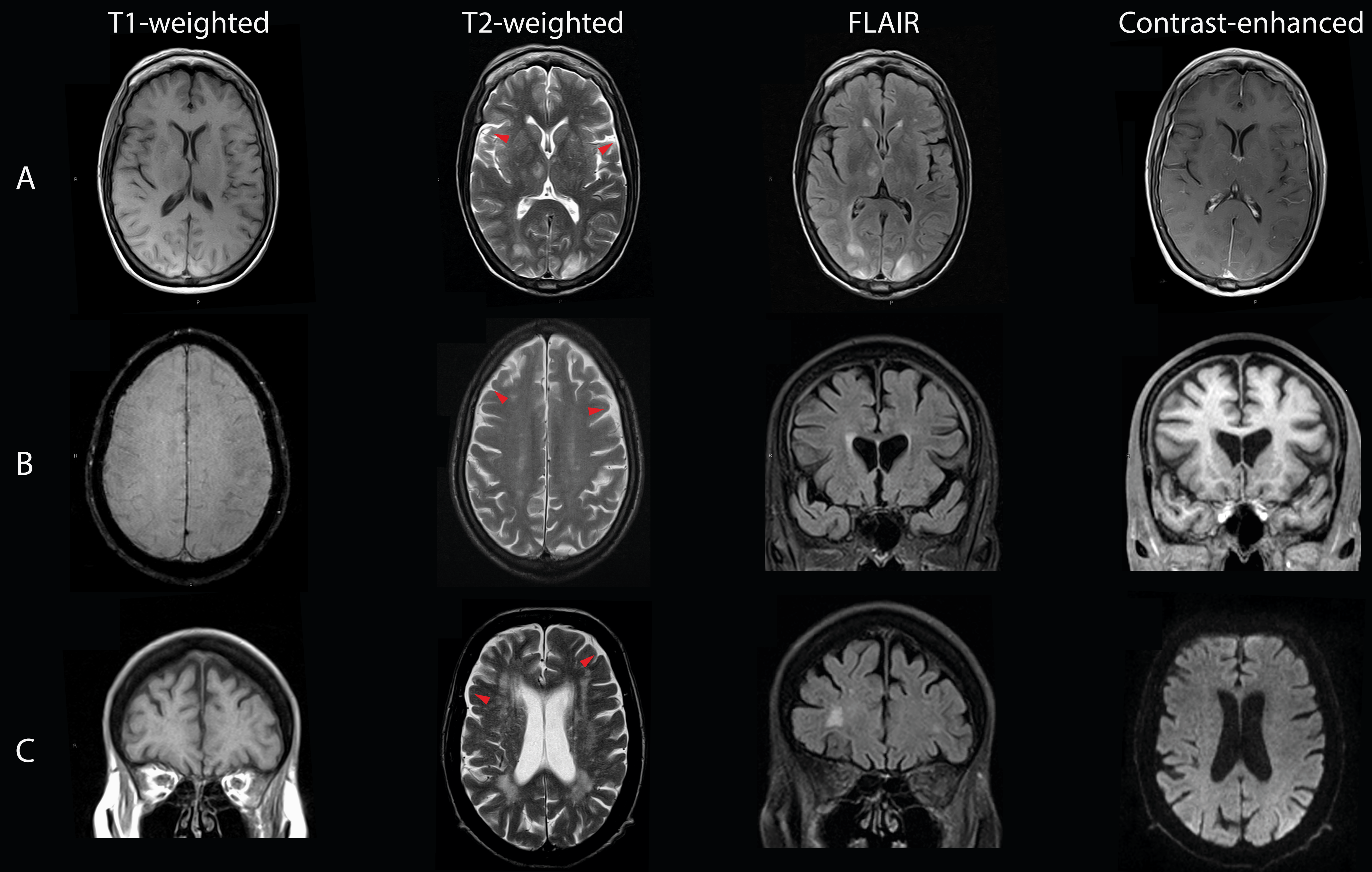

T1-weighted, T2-weighted, FLAIR and contrast enhanced in three different patients (A, B, C) all showing intermeningeal fluid effusion. Fluid is identified as low signal on T1, high signal on T2 (arrowheads), supressing on FLAIR and non-enhancing on post-gadolinium studies.

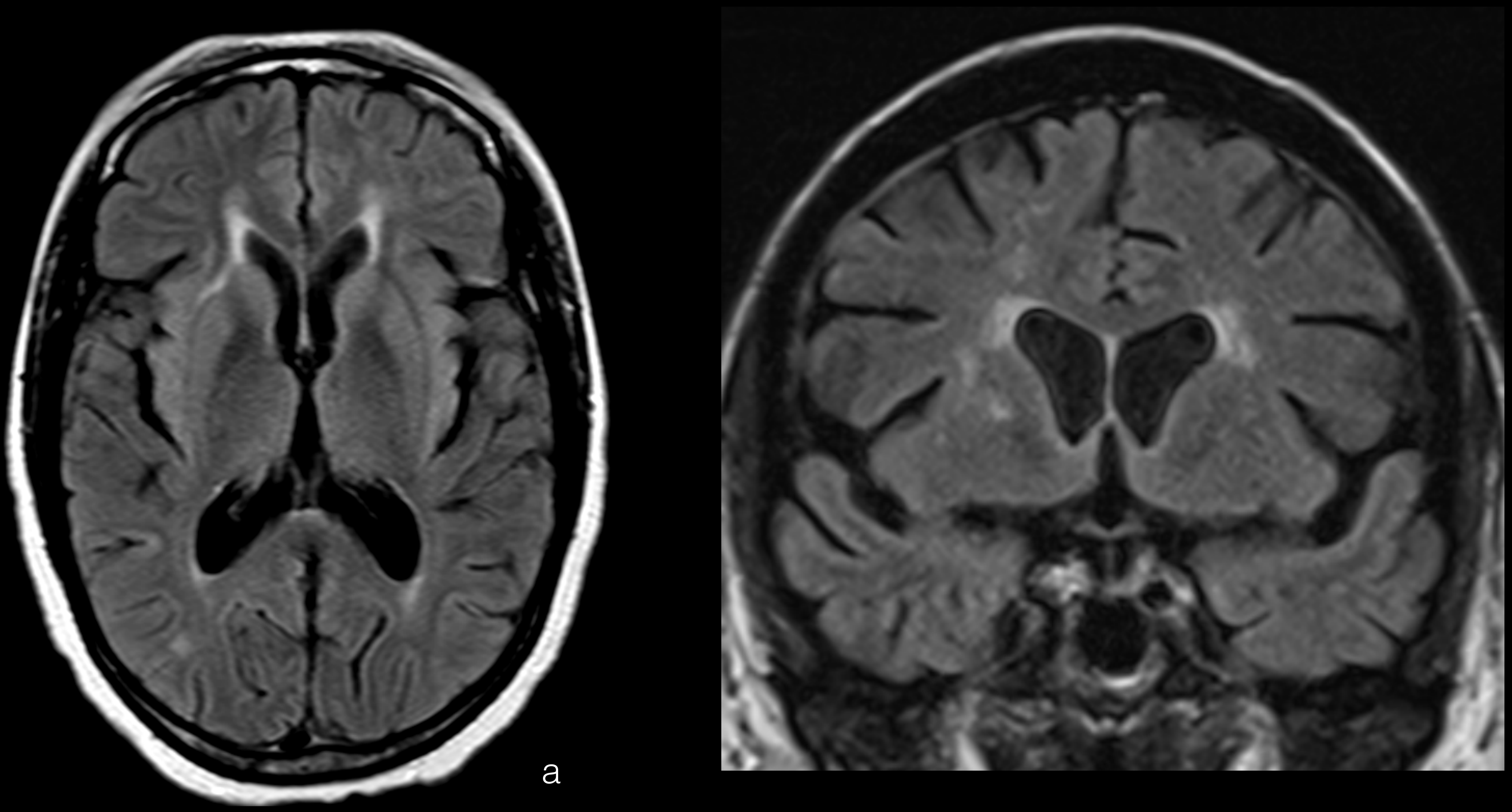

FLAIR axial (a) and coronal (b) images in two different patients showing moderate degree of small vessel disease

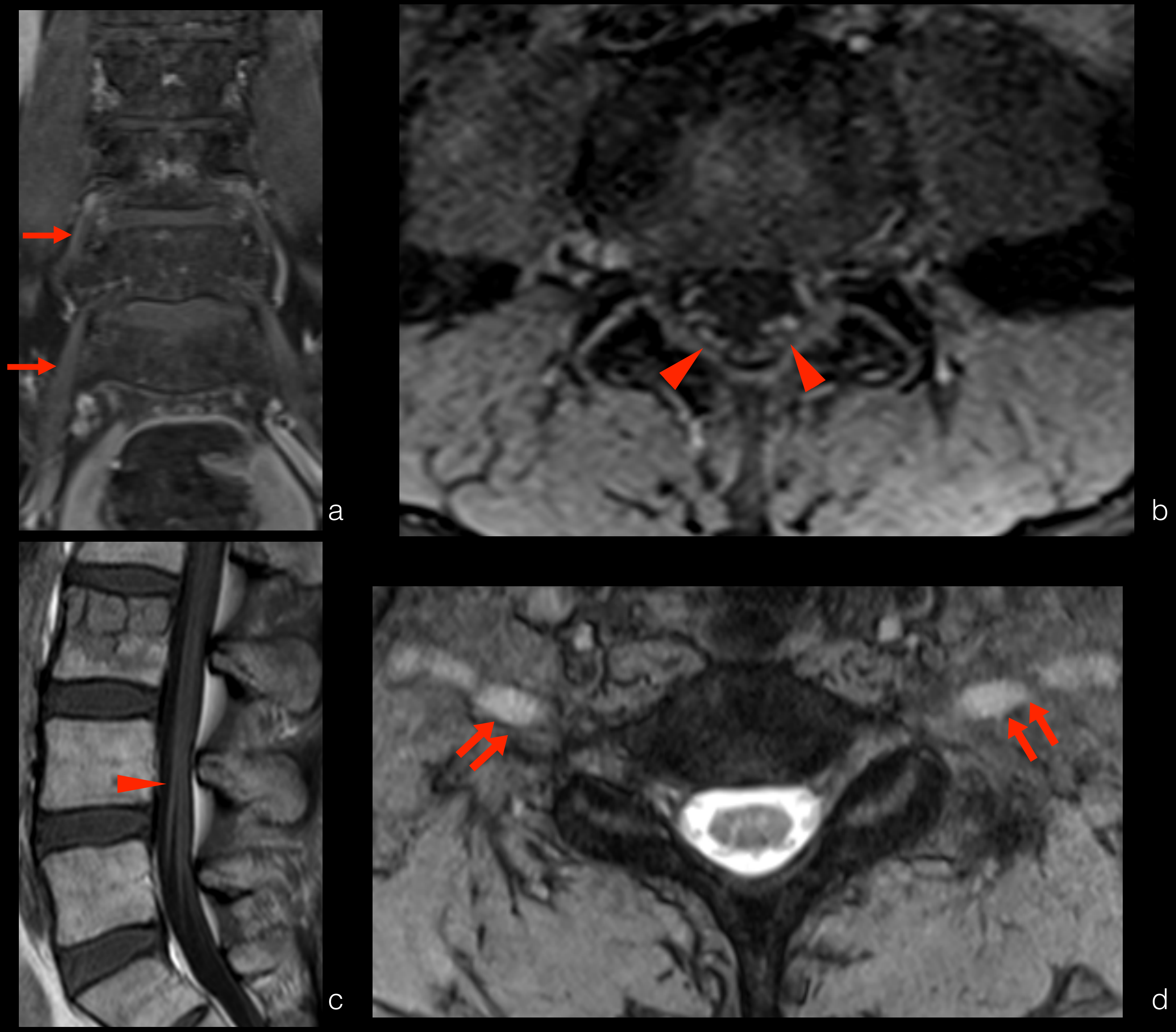

Thickened and enhancing lumbar plexus (arrows) on Post gadolinium T1w coronal image (a) and thickened brachial plexii (double arrows) on axial T2w (d) images. (b) and (c) show enhancement of cauda equina (arrow heads) in two different patients.
