# Supplementary material for: Frequent central nervous system, pachymeningeal and plexus MRI changes in POEMS syndrome
Source: J Neurol. 2019 Feb 12;266(5):1067–72. doi: 10.1007/s00415-019-09233-z (PMC6469836; doi:10.1007/s00415-019-09233-z)
Supplement: Supplementary file 2 — Supplementary material 2 (DOCX 31 KB) [file 415_2019_9233_MOESM2_ESM.docx]

**SUPPLEMENTAL MATERIAL
Frequent central nervous system, pachymeningeal and plexi MRI changes in POEMS syndrome**

## Table 1. MRI brain and spine results from individual patients with POEMS syndrome

| Demographics | | | | | | Brain MRI | | | | | | Spine MRI | |
| --- | --- | --- | --- | --- | --- | --- | --- | --- | --- | --- | --- | --- | --- |
| Age | Gender | Symptom onset | Diagnosis date | MRI date | Symptom duration (months) | Meningeal thickening | Meningeal collections | Maximal meningeal thickness (mm) | Maximal location | White matter vascular abnormality | White matter abnormality comment | Plexus thickening | Plexus thickening location |
| 72 | Male | 01/02/2009 | 01/05/2010 | 22/04/2009 | 3 | Yes | No | 2 | RIGHT FRONTAL | No |  | Yes | Brachial & Lumbar |
| 76 | Male | 01/05/2014 | 01/09/2015 | 26/04/2016 | 24 | No | No |  |  | No |  | No |  |
| 49 | Male | 01/11/2011 | 10/08/2015 | 29/07/2015 | 45 | Yes | No | 4 | RIGHT FRONTAL | No |  | No |  |
| 41 | Male | 01/02/2010 | 01/12/2010 | 08/12/2010 | 10 | Yes | No | 2 | LEFT PARIETAL | No |  | Yes | Brachial & Lumbar |
| 79 | Female | 01/05/2008 | 01/05/2010 | 21/04/2010 | 24 | Yes | No | 1 | LEFT FRONTAL | No |  | Yes | Brachial & Lumbar |
| 73 | Male | 01/01/2008 | 01/03/2009 | 10/07/2009 | 18 | Yes | No | 1 | RIGHT FRONTAL | No |  | Yes | Brachial & Lumbar |
| 72 | Male | 01/03/2008 | 01/08/2009 | 03/10/2011 | 43 | No | No |  |  | Yes | SCATTERED INCLUDING FORCEPS MAJOR | No |  |
| 59 | Male | 01/08/2011 | 01/12/2011 | 09/12/2011 | 4 | Yes | No | 5 | LEFT FRONTAL | No |  | Yes | Brachial & Lumbar |
| 78 | Male | 01/08/2013 | 01/02/2014 | 03/07/2014 | 11 | Yes | No | 1 | RIGHT FRONTAL | No |  | Yes | Brachial & Lumbar |
| 74 | Male | 01/01/2011 | 01/06/2012 | 25/08/2011 | 8 | No | No |  |  | No |  | Yes | Brachial & Lumbar |
| 62 | Male | 01/08/2011 | 01/05/2015 | 08/11/2017 | 75 | No | No |  |  | Yes | NON-SPECIFIC PERI-VENTRICULAR | Yes | Brachial only |
| 62 | Female | 01/12/2001 | 01/05/2002 | 17/08/2017 | 189 | No | No |  |  | Yes | SCATTERED NON-SPECIFIC AND CEREBELLAR INFARCT | No |  |
| 48 | Male | 01/09/2005 | 01/11/2005 | 04/01/2007 | 16 | No | No |  |  | No |  | Yes | Brachial & Lumbar |
| 71 | Female | 01/10/2006 | 01/01/2009 | 23/02/2007 | 5 | No | No |  |  | No |  | No |  |
| 67 | Male | 01/01/2011 | 01/08/2011 | 11/08/2011 | 7 | Yes | No | 2 | RIGHT POST FRONTAL | No |  | Yes | Brachial & Lumbar |
| 58 | Female | 01/06/2013 | 01/09/2014 | 03/10/2013 | 4 | Yes | No | 1 | LEFT FRONTAL | Yes | NON-SPECIFIC PERI-VENTRICULAR AND CEREBELLAR INFARCTS | No |  |
| 50 | Male | 01/01/2007 | 01/06/2013 | 20/11/2013 | 83 | Yes | No | 2 | LEFT FRONTAL | Yes | MULTI-INFARCTS | Yes | Brachial & Lumbar |
| 61 | Female | 01/10/2016 | 01/06/2017 | 02/05/2017 | 7 | Yes | No | 1 | LEFT FRONTAL | No |  | Yes | Brachial & Lumbar |
| 46 | Male | 01/01/2012 | 01/10/2013 | 29/09/2017 | 69 | Yes | No | 2 | LEFT FRONTAL | No |  | No |  |
| 51 | Male | 01/07/2011 | 01/12/2012 | 19/03/2014 | 33 | Yes | Yes | 3 | BIFRONTAL | Yes | SCATTERED NONSPECIFC T2W | Yes | Brachial & Lumbar |
| 63 | Female | 01/11/2014 | 01/02/2015 | 12/03/2017 | 28 | Yes | No | 1 | LEFT FRONTAL | Yes | MILD SVD | No |  |
| 38 | Female | 01/07/2010 | 01/06/2012 | 08/12/2017 | 89 | Yes | No | 3 | RIGHT FRONTAL | Yes | MILD SVD | No |  |
| 69 | Male | 01/09/2008 | 01/03/2009 | 29/05/2009 | 9 | Yes | No | 2 | LEFT FRONTAL | No |  | Yes | Brachial & Lumbar |
| 40 | Female | 01/10/2007 | 01/09/2009 | 05/12/2007 | 2 | Yes | No | 2 | RIGHT FRONTAL | No |  | No |  |
| 59 | Female | 01/05/2016 | 01/10/2016 | 21/12/2016 | 8 | Yes | No | 1 | RIGHT FRONTAL | No |  | Yes | Brachial & Lumbar |
| 75 | Female | 01/08/2011 | 01/03/2012 | 28/09/2017 | 74 | No | No |  |  | Yes | SOLITARY NON-SPECIFIC LEFT PERI-VENTRICULAR | Yes | Brachial only |
| 56 | Male | 01/10/2009 | 01/10/2011 | 26/10/2011 | 25 | Yes | No | 2 | BIFRONTAL | No |  | No |  |
| 71 | Male | 01/05/2017 | 01/10/2017 | 02/09/2017 | 4 | Yes | No | 2 | RIGHT FRONTAL | No |  | No |  |
| 64 | Male | 01/10/2015 | 01/11/2015 | 04/10/2017 | 24 | Yes | No | 3 | LEFT FRONTAL | Yes | MODERATE SVD | Yes | Brachial & Lumbar |
| 71 | Female | 01/11/2017 | 01/02/2018 | 25/01/2018 | 3 | No | No |  |  | Yes | BIFRONTAL, RIGHT EXTERN CAPSULAR, TEMPORAL POLES | No |  |
| 46 | Male | 01/08/2010 | 01/09/2011 | 15/05/2017 | 81 | Yes | No | 2 | RIGHT FRONTAL | Yes | GANGLIONIC AND PERI-VENTRICULAR |  |  |
| 33 | Male | 01/04/2015 | 01/11/2016 | 01/10/2015 | 6 | Yes | No | 1 | LEFT FRONTAL | Yes | MODERATE SVD |  |  |
| 54 | Female | 01/01/2001 | 01/11/2001 | 30/07/2016 | 187 | No | No |  |  | Yes | SCATTERED NON-SPECIFIC |  |  |
| 73 | Female | 01/09/2015 | 01/01/2016 | 19/09/2017 | 25 | No | No |  |  | No |  |  |  |
| 47 | Female | 01/02/2014 | 01/09/2017 | 10/08/2017 | 42 | Yes | Yes | 2 | LEFT FRONTAL | Yes | MILD SVD |  |  |
| 59 | Female | 01/09/2016 | 01/03/2017 | 12/05/2017 | 8 | Yes | Yes | 2 | LEFT FRONTAL | Yes | SCATTERED T2W PRES / VASCULAR |  |  |
| 60 | Male | 01/07/2017 | 01/12/2017 | 23/01/2018 | 7 | Yes | Yes | 4 | LEFT FRONTAL | Yes | MILD SVD |  |  |
| 36 | Male | 01/09/2017 | 01/01/2018 | 19/11/2017 | 3 | No | No |  |  | No |  |  |  |
| 60 | Male | 01/02/2016 | 01/04/2018 | 16/03/2018 | 25 | Yes | No | 2 | FALX CEREBRI | No |  |  |  |

Greyed out areas represents no relevant MRI data available for analysis. PRES, posterior reversible encephalopathy syndrome; SVD, small vessel disease (leukoaraiosis); T2W, T2 weighted MRI sequence.

## Table 2. MRI brain and spine results from individual patients with CIDP

|  | Brain MRI | | | Spine MRI | |
| --- | --- | --- | --- | --- | --- |
| Patient ID | Meningeal thickening | Meningeal collections | White matter vascular abnormality | Plexus thickening | Plexus thickening location |
| 1 | No | No | Yes | No |  |
| 2 | No | No | No | Yes | Brachial |
| 3 | No | No | Yes | No |  |
| 4 | No | No | No | No |  |
| 5 | No | No | Yes | No |  |
| 6 | No | No | No | No |  |
| 7 | No | No | No | No |  |
| 8 | No | No | No | No |  |
| 9 | No | No | Yes | No |  |
| 10 | No | No | Yes | No |  |
| 11 | No | No | Yes | Yes | Lumbosacral |
| 12 | No | No | No | Yes | Brachial |
| 13 | No | No | No |  |  |
| 14 | No | No | Yes |  |  |
| 15 | No | No | Yes |  |  |
| 16 | No | No | No |  |  |
| 17 | No | No | No |  |  |
| 18 | No | No | No |  |  |
| 19 | No | No | No |  |  |
| 20 |  |  |  | Yes | Brachial |
| 21 |  |  |  | No |  |
| 22 |  |  |  | Yes | Brachial |
| 23 |  |  |  | No |  |
| 24 |  |  |  | Yes | Brachial & Lumbosacral |
| 25 |  |  |  | Yes | Brachial |
| 26 |  |  |  | No |  |
| 27 |  |  |  | No |  |
| 28 |  |  |  | Yes | Brachial |
| 29 |  |  |  | Yes | Brachial |
| 30 |  |  |  | No |  |
| 31 |  |  |  | No |  |
| 32 |  |  |  | No |  |
| 33 |  |  |  | No |  |
